# Pathogens Associated With Linear Growth Faltering in Children With Diarrhea and Impact of Antibiotic Treatment: The Global Enteric Multicenter Study

**DOI:** 10.1093/infdis/jiab434

**Published:** 2021-09-16

**Authors:** Dilruba Nasrin, William C Blackwelder, Halvor Sommerfelt, Yukun Wu, Tamer H Farag, Sandra Panchalingam, Kousick Biswas, Debasish Saha, M Jahangir Hossain, Samba O Sow, Robert F B Reiman, Dipika Sur, Abu S G Faruque, Anita K M Zaidi, Doh Sanogo, Boubou Tamboura, Uma Onwuchekwa, Byomkesh Manna, Thandavarayan Ramamurthy, Suman Kanungo, Richard Omore, John B Ochieng, Joseph O Oundo, Sumon K Das, Shahnawaz Ahmed, Shahida Qureshi, Farheen Quadri, Richard A Adegbola, Martin Antonio, Inacio Mandomando, Tacilta Nhampossa, Quique Bassat, Anna Roose, Ciara E O’Reilly, Eric D Mintz, Usha Ramakrishnan, Helen Powell, Yuanyuan Liang, James P Nataro, Myron M Levine, Karen L Kotloff

**Affiliations:** 1 Center for Vaccine Development and Global Health, University of Maryland School of Medicine, Baltimore, Maryland, USA; 2 Department of Medicine, University of Maryland School of Medicine, Baltimore, Maryland, USA; 3 Centre for Intervention Science in Maternal and Child health (CISMAC) and Centre for International Health, University of Bergen, Bergen; 4 Cluster for Global Health, Division for Health Services, Norwegian Institute of Public Health, Oslo, Norway; 5 Department of Veterans Affairs, Cooperative Studies Program Coordinating Center, Perry Point, MD, USA; 6 Medical Research Council Unit The Gambia at London School of Hygiene and Tropical Medicine, Fajara, The Gambia; 7 Centre pour le Développement des Vaccins, Bamako, Mali; 8 Global Disease Detection Division, Kenya Office of the US Centers for Disease Control and Prevention, Nairobi, Kenya; 9 National Institute of Cholera and Enteric Diseases, Kolkata, India; 10 International Centre for Diarrhoeal Disease Research, Mohakhali, Dhaka, Bangladesh; 11 Department of Paediatrics and Child Health, the Aga Khan University, Karachi, Pakistan; 12 Kenya Medical Research Institute/Center for Global Health Research (KEMRI-CGHR), Kisumu, Kenya; 13 Centro de Investigação em Saúde de Manhiça (CISM), Maputo, Mozambique; 14 Institució Catalana de Recerca i Estudis Avançats (ICREA), Barcelona, Spain; 15 ISGlobal, Hospital Clínic - Universitat de Barcelona, Barcelona, Spain; 16 Department of Pediatrics, University of Maryland School of Medicine, Baltimore, Maryland, USA; 17 Division of Foodborne, Waterborne, and Environmental Diseases, US Centers for Disease Control and Prevention, Atlanta, GA, USA; 18 Hubert Department of Global Health, Rollins School of Public Health, Emory University, Atlanta, Georgia, USA; 19 Department of Epidemiology and public health, University of Maryland School of Medicine, Baltimore, Maryland, USA

**Keywords:** Diarrhea, pathogens, growth faltering, stunting, children, antibiotics

## Abstract

**Background:**

The association between childhood diarrheal disease and linear growth faltering in developing countries is well described. However, the impact attributed to specific pathogens has not been elucidated, nor has the impact of recommended antibiotic treatment.

**Methods:**

The Global Enteric Multicenter Study enrolled children with moderate to severe diarrhea (MSD) seeking healthcare at 7 sites in sub-Saharan Africa and South Asia. At enrollment, we collected stool samples to identify enteropathogens. Length/height was measured at enrollment and follow-up, approximately 60 days later, to calculate change in height-for-age *z* scores (ΔHAZ). The association of pathogens with ΔHAZ was tested using linear mixed effects regression models.

**Results:**

Among 8077 MSD cases analyzed, the proportion with stunting (HAZ below −1) increased from 59% at enrollment to 65% at follow-up (*P* < .0001). Pathogens significantly associated with linear growth decline included *Cryptosporidium* (*P* < .001), typical enteropathogenic *Escherichia coli* (*P* = .01), and untreated *Shigella* (*P* = .009) among infants (aged 0–11 months) and enterotoxigenic *E. coli* encoding heat-stable toxin (*P* < .001) and *Cryptosporidium* (*P* = .03) among toddlers (aged 12–23 months). *Shigella-*infected toddlers given antibiotics had improved linear growth (*P* = .02).

**Conclusions:**

Linear growth faltering among children aged 0–23 months with MSD is associated with specific pathogens and can be mitigated with targeted treatment strategies, as demonstrated for *Shigella*.

Diarrheal disease is associated with linear growth faltering among young children [1]. Community-based studies in low-resource settings demonstrate an increasing risk of stunting at age 24 months with each diarrheal episode and each day of diarrhea before that age [2]. In turn, stunting is a risk factor for poor health and development [3]. Even mild stunting predicts an increased risk of death during the first 2 years of life [4].

Although most acute diarrhea is caused by infection, few studies have elucidated the impact of specific pathogens on growth, and a limited array of pathogens has been examined [5–8]. Moreover, trials designed to evaluate the efficacy of pathogen-specific treatment have used short-term clinical and bacteriologic cure as end points [9, 10], while the impact on growth has not been characterized.

The Global Enteric Multicenter Study (GEMS) is a prospective, matched case-control study of the burden, etiology, and adverse clinical outcomes of moderate to severe diarrhea (MSD) in children aged 0–59 months in sub-Saharan Africa and South Asia. GEMS found an association between MSD and linear growth faltering [11, 12]. In this study, we assessed pathogen-specific associations and whether antibiotic treatment of *Shigella* dysentery, according to World Health Organization (WHO) recommendations, improved growth outcomes.

## MATERIALS AND METHODS

### Study Design and Participants

For 36 months at each site, children aged 0–59 months were enrolled into GEMS at sites in Basse (The Gambia), Bamako (Mali), Manhiça (Mozambique), Siaya County (Kenya), Kolkata (India), Mirzapur (Bangladesh), and Karachi, Bin Qasim town (Pakistan), according to published methods [13–15]. To be eligible for enrollment, a child had to reside in the site’s demographic surveillance area, seek care at a study health center with diarrhea (defined as *≥*3 abnormally loose stools in the previous 24 hours) that began in the previous 7 days, and meet ≥1 of the following criteria for MSD: sunken eyes, decreased skin turgor, visible blood in stool, or a clinician recommendation for intravenous rehydration or hospitalization. We aimed to enroll approximately 220 eligible children per site per year into each of 3 age groups: infants (0–11 months), toddlers (12–23 months), and young children (24–59 months). Children were eligible for reenrollment if a new episode of MSD developed after the 60-day follow-up visit [13]. For this report, only children with MSD were included. Although GEMS included a case-control component described elsewhere [12, 13], for this paper, only cases (children with MSD) were analyzed.

### Data Collection

During the enrollment visit at the health center, all children underwent a standardized clinical assessment and anthropometric measurements and provided a stool sample to identify enteropathogens [13, 15]. Stool collection, transport, and pathogen identification methods have been described elsewhere [16]. Management of diarrhea at the health center, including antibiotic use, was documented. Approximately 60 days after enrollment (49–91 days), participants were visited at home for assessment of vital status and repeated anthropometric measurements.

### Anthropometric Measurements

We measured standing height for children ≥2 years old, and recumbent length for younger children and those unable to stand unassisted, thrice to the nearest 0.1 cm, using a ShorrBoard measuring board and following a 2-person standardized measurement procedure [17]. The median of the 3 measurements was used to calculate the height-for-age *z* score (HAZ) [18, 19].

Before study initiation, staff at each site underwent anthropometry training followed by a standardization exercise to calculate intrarater and interrater (trainee vs instructor) variability [13]. Trainees who exceeded the acceptable measurement error (0.5 cm) at least half the time did not undertake field activities until retrained and deemed competent. A proficient member of each local team provided training and standardization exercises for newly hired staff and refresher training for existing staff every 4–6 months. The core team visited each site approximately twice annually to train, observe field activities, and review standardization results.

The study was approved by ethics committees at the University of Maryland, Baltimore, and each field site. Informed consent was obtained from the parent or guardian of each child before study procedures were performed.

### Statistical Methods

#### Variable Definitions

Age was measured on a continuous scale, and analyses were stratified by age group. The HAZ was calculated at enrollment and follow-up, using the median of the 3 length/height measurements and age, according to WHO standards [19]. We defined stunting as HAZ below −1 and the degree of stunting as mild (HAZ <−1 and >/=−2), moderate (HAZ <−2 and >/=−3), or severe (HAZ<−3), at enrollment and follow-up.

#### Data Analysis

This analysis included children with MSD with both enrollment and follow-up measurements; implausible or inconsistent measurements between enrollment and follow-up were excluded (Supplementary Figure 1).

The primary outcome is change in linear growth (change in HAZ [ΔHAZ]), which was calculated for each child as the difference in HAZ from enrollment to follow-up. A negative change in linear growth is deemed growth faltering. We initially compared enrollment and follow-up HAZ within each age group (and study site), using a 1-sample *t* test for ΔHAZ and adjusted for individuals with multiple episodes of MSD during the study period (SAS proc surveymeans t test with CLUSTER statement). we compared the presence and degree of stunting at enrollment and follow-up, using a Wilcoxon signed-rank test.

Pathogens were tested for association with change in linear growth within each age group. We limited the analysis to pathogens that were (1) significantly associated with MSD in ≥4 sites [12] and/or (2) associated with increased risk of dying in a pooled analysis of children with MSD at all sites [12]. For the first criterion, we included, for all age groups, rotavirus, *Cryptosporidium*, *Shigella*, and enterotoxigenic *E. coli* (ETEC) encoding heat-stable toxin (ST-ETEC) with or without targets for heat-labile enterotoxin, and for the 2 youngest groups, adenovirus serotypes 40 and 41. For the second criterion, we also included typical enteropathogenic *E. coli* (tEPEC) in the analyses involving infants [12]. We used a linear mixed effects regression model (LMM) to examine the association of each of the above-named pathogens individually with change in linear growth among children with MSD by age group. In addition to the pathogen, the model also included HAZ at enrollment, age at enrollment in months, duration to follow-up in days, and study site.

Treatment of *Shigella* with a WHO-recommended antibiotic (ciprofloxacin, third-generation cephalosporins, azithromycin, or pivmecillinam) [20] could modify any potential association of *Shigella* with linear growth. We therefore examined the need for an interaction term between the presence and absence of *Shigella* with or without antibiotic treatment. The interaction term was retained if the associated *P* value was <.10. Pathogens associated (*P* < .10) with ΔHAZ in the individual models were combined into a single age-stratified model, which included the same confounding variables described previously for the individual models. Unless otherwise stated, associations were considered statistically significant at *P* < .05. SAS software, version 9.4 (SAS Institute), was used for all summary statistics and associated tests; STATA/SE software, version 17, was used to fit the LMMs.

## RESULTS

### Study Participants

Between 1 December 2007 and 3 March 2011, a total of 8077 MSD episodes in 7545 children were analyzed: 3408 were aged 0–11 months, 2741 aged 12–23 months, and 1928 aged 24–59 months. There were 1362 episodes excluded from analysis owing to missing measurements (63.2%), implausible values (17.8%), death before follow-up (14.0%), and follow-up visit outside the acceptable time window (5.0%) (Supplementary Figure 1). Children with excluded and included episodes had similar demographic features (Supplementary Table 1), except those excluded because of death had significantly lower enrollment HAZ than included children in 14 of 21 comparisons (3 age groups at 7 sites).

Mean enrollment HAZ in each age group, at every site, was <0 and became more negative with increasing age (Supplementary Table 2), while the proportion with stunting at enrollment increased with age (Table 1). A total of 58.7% of children were stunted at enrollment (mild stunting in 32.2%, moderate in 18.0%, and severe in 8.5%).

**Table 1.** Degree of Stunting in Children With Moderate to Severe Diarrhea at Enrollment and at Follow-up, by Age Group

	Degree of Stunting at Follow-up, No. (%)^a^				
Degree of Stunting at Enrollment by Age Group	Total	None	Mild	Moderate	Severe
Age 0–11 mo					
Total	3408	1414 (41.5)	1093 (32.1)	616 (18.1)	285 (8.3)
None	1727 (50.7)	1302 (75.4)	391 (22.6)	32 (1.9)	2 (0.1)
Mild	1049 (30.8)	104 (9.9)	640 (61.0)	291 (27.7)	14 (1.3)
Moderate	435 (12.7)	8 (1.8)	57 (13.1)	257 (59.1)	113 (26.0)
Severe	197 (5.8)	0	5 (2.5)	36 (18.3)	156 (79.2)
Age 12–23 mo					
Total	2741	838 (30.6)	951 (34.7)	591 (21.6)	361 (13.2)
None	998 (36.4)	774 (77.6)	222 (22.2)	2 (0.2)	0
Mild	911 (33.2)	63 (6.9)	654 (71.8)	191 (21.0)	3 (0.3)
Moderate	556 (20.3)	1 (0.2)	74 (13.3)	374 (67.3)	107 (19.2)
Severe	276 (10.1)	0	1 (0.4)	24 (8.7)	251 (90.9)
Age 24–59 mo					
Total	1928	582 (30.2)	647 (33.6)	457 (23.7)	242 (12.6)
None	612 (31.7)	528 (86.3)	84 (13.7)	0	0
Mild	642 (33.3)	54 (8.4)	509 (79.3)	79 (12.3)	0
Moderate	462 (24.0)	0	54 (11.7)	358 (77.5)	50 (10.8)
Severe	212 (11.0)	0	0	20 (9.4)	192 (90.6)

^a^Follow-up visits were approximately 60 days after enrollment (range, 49–91 days). Stunting was significantly more severe at follow-up in all 3 age groups (*P* < .001; Wilcoxon signed-rank test). The degree of stunting was defined according to height-for-age z scores (HAZ), No stunting defined as HAZ ≥ −1, with mild stunting defined as HAZ between <−1 and >/+−2, moderate stunting as HAZ between <−2 and ≥−3, and severe stunting as HAZ below <−3.

The proportion of children with stunting was significantly higher at follow-up than at enrollment in every age group (Table 1 and Supplementary Table 2), with a difference of 9.2% in infants, 5.8% in toddlers, and 1.5% in young children. A total of 64.9% of children were stunted at follow-up (mild stunting in 33.3%, moderate in 20.6%, and severe in 11.0%).

### Pathogens Associated With Linear Growth Faltering

In the individual-pathogen analyses for infants, the presence of *Cryptosporidium* or tEPEC was significantly associated with a greater decline in linear growth than in those without either pathogen (difference in ΔHAZ, −0.09 for *Cryptosporidium* [95% confidence interval (CI), −.14 to −.04; *P* < .001] and −0.08 for tEPEC [−.15 to −.02; *P* = .01]) (Table 2). In addition, the interaction between *Shigella* and WHO-recommended antibiotics was statistically significant (*P* = .01). *Shigella* episodes not treated with antibiotics resulted in a greater decline in linear growth compared with treated episodes (difference in ΔHAZ, −0.16 [95% CI, −.29 to −.03]; *P* = .02), while *Shigella* episodes treated with antibiotics were not associated with positive or negative linear growth. These 3 pathogens, including the interaction term, were included in a final fully adjusted model. Rotavirus and ST-ETEC were not associated with linear growth faltering among infants.

**Table 2.** Change in Linear Growth Between Enrollment and Follow-up in Children From 7 Global Enteric Multicenter Study Sites, by Age Group, Using Individual- and Multiple-Pathogen Models

		Individual-Pathogen Model		Multiple-Pathogen Model	
Pathogen by Age Group	Episodes With pathogen, No. (%)	Difference in ΔHAZ (95% CI)	*P* Value	Difference in ΔHAZ (95% CI)	*P* Value
Age 0–11 mo (n = 3408)					
* Cryptosporidium*	525 (15.4)	−0.09 (−.14 to −.04)	<.001	−0.09 (−.14 to −.04)	<.001
* Shigella,* not treated with antibiotic	72 (2.1)	−0.16 (−.29 to −.03)	.02	−0.17 (−.31 to −.04)	.009
* Shigella,* treated with antibiotic	93 (2.7)	0.05 (−.07 to .17)	.38	0.05 (−.07 to .17)	.41
* *Typical EPEC	304 (8.9)	−0.08 (−.15 to −.02)	.01	−0.08 (−.15 to −.02)	.01
* *Rotavirus	859 (25.2)	0.02 (−.02 to .06)	.40	…	…
* *ST-ETEC	203 (6.0)	0.006 (−.07 to .08)	.89	…	…
* *Adenovirus 40/41	104 (3.0)	−0.06 (−.17 to .05)	.27	…	…
Age 12–23 mo (n = 2741)					
* Cryptosporidium*	312 (11.4)	−0.05 (−.09 to −.003)	.04	−0.05 (−.09 to −.005)	.03
* Shigella*, not treated with antibiotic	159 (5.8)	−0.06 (−.12 to .006)	.08	−0.06 (−.12 to .001)	.054
* Shigella*, treated with antibiotic	282 (10.3)	0.07 (0.01 to 0.13)	.02	0.06 (.009 to .13)	.02
* *Rotavirus	492 (18.0)	−0.04 (−.07 to .002)	.06	…	…
* *ST-ETEC	199 (7.3)	−0.12 (−.17 to −.06)	<.001	−0.12 (−.17 to −.06)	<.001
* *Adenovirus 40/41	76 (2.8)	0.008 (−.08 to .09)	.86	…	…

Abbreviations: ΔHAZ, change in height-for-age *z* scores; CI, confidence interval; ST-ETEC, enterotoxigenic *Escherichia coli* encoding heat-stable toxin.

Among toddlers, *Cryptosporidium* was associated with a greater decline in linear growth compared with those without *Cryptosporidium* (difference in ΔHAZ, −0.05 [95% CI, −.09 to −.003]; *P* = .04) (Table 2), with the difference in ΔHAZ very similar to that in infants. The presence of rotavirus or ST-ETEC also resulted in a greater decline in linear growth (difference in ΔHAZ, −0.04 for rotavirus [95% CI, −.07 to −.002; *P* = .06] and −0.12 for ST-ETEC [95% CI, −.17 to −.06; *P* < .001]). The result for rotavirus was not statistically significant but met our criteria for inclusion in the final adjusted model. The interaction term between *Shigella* and antibiotic treatment was significant (*P* = .003), and antibiotic treatment improved linear growth in toddlers with shigellosis (difference in ΔHAZ 0.07 [95% CI, .01 to .13]; *P* = .02). *Shigella* not treated with antibiotics resulted in further declines in linear growth (difference in ΔHAZ, −0.06 [95% CI, −.12 to .006]; *P* = .08). Adenovirus 40/41 was not associated with linear growth among children with MSD aged 12–23 months.

Each of *Cryptosporidium*, rotavirus, ST-ETEC, and the interaction between *Shigella* and antibiotic treatment were included in a final model; however, rotavirus was subsequently excluded from the final model because the association was not statistically significant. For those pathogens remaining in the final model, the results of this pathogen-adjusted analysis were very similar to those of the individual-pathogen analyses (Table 2). None of the individual pathogens were associated with linear growth faltering in the oldest age group (24−59 months).

### Antibiotic Prescribing Practices and Susceptibility Patterns

**Figure 1.**
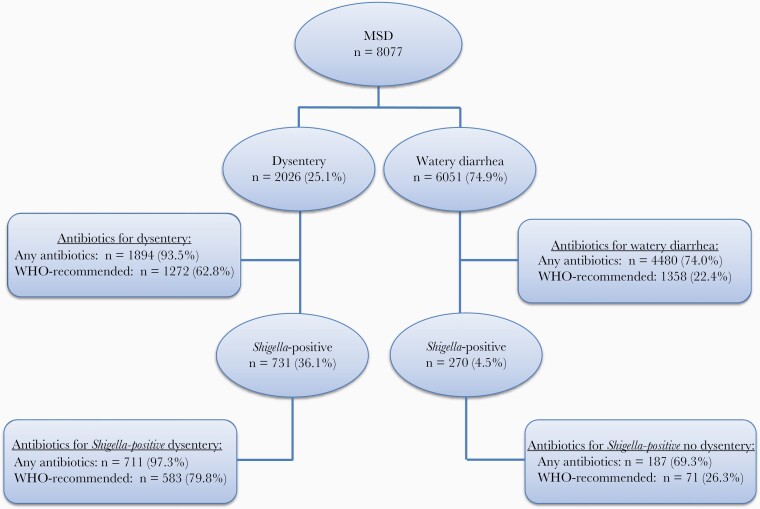
Distribution of diarrhea episodes included in the analysis according to the presence of dysentery, *Shigella* isolation, and antibiotic treatment. Abbreviation: MSD, moderate to severe diarrhea; WHO, World Health Organization.

In an ad hoc analysis, we further explored the observed association between linear growth faltering and failure to treat *Shigella* with antibiotics recommended by WHO for dysentery. For context, we first examined antibiotic prescribing practices for *Shigella* dysentery (Figure 1 and Supplementary Table 3) and the susceptibility patterns of offending strains.

Of all MSD episodes in children, 2026 (25.1%) had dysentery; 1894 (93.5%, range 79.9% [Pakistan] to 99.6% [Bangladesh]) were prescribed antibiotics. The most common antibiotics were ciprofloxacin, trimethoprim-sulfamethoxazole, and metronidazole (59.9%, 22.6%, and 11.6%, respectively). A WHO-recommended antibiotic was prescribed for 1272 dysentery episodes (62.8%) and for 583 *Shigella*-positive dysentery episodes (79.8%) (Figure 1 and Supplementary Table 3).

There was considerable regional diversity in antibiotic prescribing practices. A WHO-recommended antibiotic was prescribed for 614 of the 621 *Shigella* dysentery episodes in Asia (98.9%), but for only 20 of the 110 episodes at the 4 African sites (18.1%). Ciprofloxacin was the most prescribed antibiotic at the Asian sites, including India (77.8%), Bangladesh (86.5%), and Pakistan (78.9%), but it was a rare choice in Africa. At the African sites, the most commonly prescribed antibiotic was trimethoprim-sulfamethoxazole in The Gambia (60.0%), Mali (60.0%), and Kenya (40.5%), and nalidixic acid in Mozambique (87.5%).

Virtually all (*≥*99%) *Shigella* strains were susceptible to ciprofloxacin in The Gambia, Mali, Mozambique, Kenya, and Pakistan, compared with 87.0% in Bangladesh and only 35.4% in India. At the African sites, >80% of *Shigella* isolates were resistant to trimethoprim-sulfamethoxazole, while 85% were susceptible to nalidixic acid in Mozambique.

### Impact of Antibacterial Treatment of Shigellosis on Linear Growth

*Shigella* was cultured in 731 of the dysentery episodes (36.1%) and 270 watery diarrhea episodes (4.5%) (Figure 1). A WHO-recommended antibiotic was prescribed for 583 *Shigella*-positive dysentery episodes (79.8%) and 71 *Shigella*-positive watery diarrhea episodes (26.3%) (Figure 1). Analysis of the impact of antibiotic treatment of shigellosis on linear growth was performed separately for *Shigella-*positive watery diarrhea and dysentery (Table 3).

**Table 3.** Linear Growth After Episodes of *Shigella-*Positive Dysentery or Watery Diarrhea, According to Whether World Health Organization–Recommended Antibiotic Treatment Was Prescribed

	Prescription Status by Age Group^a^					
	0–11 mo		12–23 mo		24–59 mo	
*Shigella*-Positive Diarrhea or Dysentery	Prescribed	Not Prescribed	Prescribed	Not Prescribed	Prescribed	Not Prescribed
Watery diarrhea	n = 11	n = 45	n = 29	n = 92	n = 31	n = 62
ΔHAZ (95% CI)^b^	−0.40 (−.63 to −.17)	−0.44 (−.57 to −.31)	−0.16 (−.36 to .04)	−0.25 (−.34 to −.16)	−0.15 (−.27 to −.02)	−0.13 (−.21 to −.05)
Dysentery	n = 82	n = 26	n = 253	n = 67	n = 248	n = 55
ΔHAZ (95% CI)^b^	−0.21 (−.28 to −.13)	−0.47 (−.72 to −.23)	−0.10 (−.18 to .03)	−0.37 (−.48 to −.26)^c^	−0.12 (−.15 to −.08)	−0.14 (−.28 to −.009)

Abbreviations: ΔHAZ, change in height-for-age *z* scores; CI, confidence interval.

^a^Prescription of a World Health Organization–recommended antibiotic for dysentery (ciprofloxacin, third-generation cephalosporin, azithromycin, or pivmecillinam).

^b^Assessed using linear regression, controlling for age, site, enrollment height-for-age *z* score (HAZ), and days to follow-up visit.

^c^ *P* = .03.

In toddlers, whose incidence of *Shigella*-positive MSD was almost double that of the other 2 age groups [12], an approximately 4-fold reduction in linear growth faltering was observed among *Shigella*-positive dysentery treated with WHO-recommended antibiotics, compared with untreated children (ΔHAZ, −0.10 [95% CI, −.18 to .03] vs −0.37 [−.48 to −.26]; *P* = .03). A similar trend was observed among infants with *Shigella*-positive dysentery and toddlers with *Shigella*-positive watery diarrhea, but the differences did not reach statistical significance with the small sample sizes (Table 3).

To address whether antibiotics exert a nonspecific growth-promoting effect on children with diarrhea, we compared ΔHAZ among children with MSD associated with rotavirus (n = 1493) or *Cryptosporidium* (n = 945) who were offered ciprofloxacin, third-generation cephalosporins, azithromycin, or pivmecillinam with those who were not offered these antibiotics. Overall, 33.0% of rotavirus-positive and 32.0% of *Cryptosporidium*-positive children received 1 of these antibiotics, but no significant associations with linear growth were observed.

## DISCUSSION

We previously demonstrated that among children 0–59 months of age living in low-resource settings in South Asia and sub-Saharan Africa, an episode of MSD was associated with an increased risk of stunting over the ensuing 2–3 months. Now we present findings involving a broad array of pathogens indicating that 4 pathogens (*Cryptosporidium*, tEPEC, *Shigella*, and ST-ETEC) exert the largest negative effect on linear growth among infants and toddlers. We found that the risk associated with *Shigella* was largely limited to episodes not treated with WHO-recommended antibiotics.

To our knowledge, this is the first study to demonstrate that antibiotic treatment of dysentery according to WHO guidelines significantly ameliorates the linear growth impairment associated with *Shigella* infection, and in toddlers also augments growth. Previous clinical trials of children with shigellosis examined the short-term benefits of antibiotics (resolution of diarrheal disease symptoms and fecal shedding) but did not evaluate the impact on linear growth faltering, widely believed to be a proxy for mortality rate and poor health outcomes in the longer term. We observed a statistically significant improvement in linear growth among children with *Shigella*-positive dysentery with WHO-recommended antibiotic in the 12–23-month age group, and a similar trend among infants with *Shigella*-positive dysentery and toddlers with *Shigella*-positive watery diarrhea that did not reach statistical significance.

Regional differences in executing WHO guidelines were apparent. Although most Asian sites administered recommended first-line therapy with ciprofloxacin, 20% of episodes were missed, and the escalating prevalence of ciprofloxacin resistance threatens the efficacy of recommended treatment, particularly in India. The African sites provided treatment to only 20% of *Shigella*-positive dysentery episodes, but in most cases *Shigella* was resistant to the antibiotic chosen (trimethoprim-sulfamethoxazole). The prevalence of resistance to trimethoprim-sulfamethoxazole precludes its routine use unless supported by local susceptibility patterns.

Our findings corroborate and expand results of studies in Peru [6] and Guinea Bissau [7], which suggested that *Cryptosporidium* infection in infancy imparted a lasting adverse effect on linear growth. Our group previously reported that this risk period extends to include the second year of life, when *Cryptosporidium* was strongly associated with MSD at all GEMS sites, regardless of HIV prevalence [12], and was associated with death during the approximately 60 days after an MSD episode in toddlers. Our current findings suggest that the impact of *Cryptosporidium* on mortality rates may be linked to its considerable nutritional insult, as measured by a negative ΔHAZ. Strategies for point-of-care diagnosis and identification of appropriate therapeutic agents for case management of cryptosporidiosis in low-resource settings are needed and should be evaluated for their impact on growth, and if possible, survival.

Our findings also demonstrate an association between ST-ETEC diarrhea and linear growth faltering during the second year of life. Previous studies of the relationship between ST-ETEC and stunting have conflicting results. One study of children aged 3–48 months in rural Bangladesh demonstrated a significant association between the percentage of days with ETEC diarrhea during 60-day intervals and failure to gain weight, but not an association with impeded linear growth [5]. Another study involving a birth cohort followed up for 2 years in urban Bangladesh found that stunted or undernourished children aged 12–24 months were significantly more likely to have experienced ETEC diarrhea than those who were not stunted [21].

Our analysis differs from previous studies in that it distinguishes ST-ETEC from the less virulent ETEC pathotype encoding only LT, and we limited enrollment to more clinically severe forms of diarrheal illness. In addition, we examined linear growth over a relevant time frame (2–3 months after MSD onset) and demonstrated the impact of ST-ETEC relative to other pathogens that were significantly associated with MSD. Nonetheless, the observed effect of ST-ETEC infection on linear growth was somewhat unexpected, since ETEC is often considered a self-limited secretory diarrhea, which, in animal models [22] and human challenge studies, caused little or no inflammation or alteration of intestinal integrity that might interfere with growth [23, 24]. Other data, however, suggest that ST-ETEC can elicit an inflammatory response involving interleukin 8 expression [25, 26]. Peruvian children younger than 2 years with ETEC infection had fecal leukocytes in their diarrheal stools [27].

Several limitations of this study are worthy to mention. First, unmeasured events between enrollment and follow-up may have influenced growth, so it is notable that children with MSD grew significantly less during follow-up than their matched controls, despite having comparable HAZ at enrollment [12]. Second, it is possible that the detrimental effects of growth faltering after MSD can be overcome by catch-up growth. Even if that were the case, the 2–3 months after MSD onset was a particularly vulnerable period when mortality rates among children with MSD were 8_._5-fold higher than among matched controls [12]. We recognize that observational studies are suboptimal for evaluating the impact of antibiotics against *Shigella-*associated linear growth faltering; however, the inclusion of objective end points (height/length, predefined diarrhea and dysentery, and findings of fecal microbiology) mitigate these concerns. Finally, we used directly observed inpatient administration of antibiotics and/or prescription of antibiotics as a proxy for antibiotic use and were unable to measure compliance.

In conclusion, our findings suggest that prevention or treatment of infection with 4 pathogens (*Cryptosporidium*, ST-ETEC, tEPEC, and *Shigella*) may reduce the burden of linear growth faltering in children. The adverse effects of *Shigella* were mitigated by administration of WHO-recommended antibiotics; however, these antibiotics were prescribed suboptimally in only 63% of dysentery episodes, and in several sites antibiotic practices did not match local susceptibility patterns. We recognize that antibiotic use has been associated with increasing resistance of Shigella to antibiotics, which globally could leave few options for effective therapy [28]. Accordingly, WHO has declared antibiotic-resistant *Shigella* to be a serious threat [29]. It seems prudent that for benefit to exceed risk, treatment of shigellosis should be judicious, guided by susceptibility data when possible, and directed toward individuals at risk for severe disease or complications. Because stunting is prevalent worldwide and directly associated with poor outcomes, interventions with a relatively small but significant effect have the potential to benefit many children’s lives.

## Supplementary Material

jiab434_suppl_Supplementary_Materials

## Notes

***Acknowledgments.*** We thank the families who participated in these studies, the project field staff for their hard work and dedication, and the physicians and administration at every site who generously provided facilities for the conduct of the study. We also thank Irwin J. Shorr for anthropometry training and development of anthropometry training materials, Annemieke Van ejik and Lynette Berkeley for their role in clinical and microbiologic data collection, Leslie Jamka for reviewing the manuscript, and Rebecca J. Stoltzfus for her advice on analysis and review of the manuscript.

***Author contributions.*** M. M. L. conceived the project and acquired the grant funds. J. P. N., M. M. L., and K. L. K. designed the protocol. D. N., with W. C. B., H. S., Y. W., A. R., U. R., H. P., Y. L., and K. L. K., did the statistical analysis. D. N., T. H. F., S. P., D. Saha, M. J. H., S. O. S., R. F. B., D. Sur, A. S. G. F., A. K. M. Z., R. A. A., C. E. O. R., E. D. M., J. P. N., and K. L. K. planned and supervised the study. D. Sanogo, S. K., R. O., S. K. D., S. A., F. Q., T. N., and Q. B. coordinated clinical data collection; B. T., T. R., J. B. O., J. O. O., S. Q., M. A., I. M., did the laboratory assays; and K. B., M. J. H., U. O., and B. M. participated in data management. D. N., W. C. B., Y. W., T. H. F., J. P. N., M. M. L., and K. L. K. had full access to all the data in the study and did data analysis. D. N. and K. L. K. wrote the manuscript with input from all authors and had final responsibility for the decision to submit for publication. All authors reviewed the draft and approved the decision to submit for publication.

***Financial support.*** This work was supported by the Bill & Melinda Gates Foundation.

***Potential conflicts of interest.*** All authors: No reported conflicts. All authors have submitted the ICMJE Form for Disclosure of Potential Conflicts of Interest. Conflicts that the editors consider relevant to the content of the manuscript have been disclosed.
