# Supplementary material for: Pathogens Associated With Linear Growth Faltering in Children With Diarrhea and Impact of Antibiotic Treatment: The Global Enteric Multicenter Study
Source: J Infect Dis. 2021 Sep 16;224(Suppl 7):S848–55. doi: 10.1093/infdis/jiab434 (PMC8958895; doi:10.1093/infdis/jiab434)
Supplement: jiab434_suppl_Supplementary_Materials [file jiab434_suppl_Supplementary_Materials.docx]

**SUPPLEMENTAL TABLES AND FIGURES**

**Figure S1: Steps of data cleaning**

243 had implausible Δheight*:

- 133 decreased >1.5 cm
- 14 participants <6 months old at enrollment increased by:
- >8 cm at 49-60 days (n=6)
- >10 cm at 61-91 days (n=8)
- 96 participants >6 months old at enrollment increased by:
  - >4 cm at 49-60 days (n=51)
  - >6 cm at 61-91 days (n=45)

68 had follow-up visit outside 49-91 day window

- 190 died
- 861 had missing values:
- 5 lacked enrollment measure
- 852 lacked follow-up measure
- 4 lacked both measures

Included in analysis

N=8,077

Height measured at enrollment and follow-up

N=8,388

Height measured per protocol windows

N=8,320

Total MSD episodes

N=9,439

**Figure S1.** Consort diagram showing the reasons that MSD episodes were excluded in order to derive an analyzable sample for assessing the length/height for age z score (HAZ) and the change in HAZ (ΔHAZ) between enrollment and follow-up 49-91 days later. *Δheight=change in height from the enrollment to the follow-up visit. Shaded boxes depict reasons for exclusion.

**Table S1: Comparison of demographic and epidemiologic characteristics between MSD episodes included in the analysis and those excluded because of death or for other reasons**

|  | Basse,  The Gambia | | | | Bamako,  Mali | | | Manhiça, Mozambique | | | Kisumu,  Kenya | | | | Kolkata,  India | | | | Mirzapur,  Bangladesh | | | | | | Karachi (Bin Qasim Town) Pakistan | | |
| --- | --- | --- | --- | --- | --- | --- | --- | --- | --- | --- | --- | --- | --- | --- | --- | --- | --- | --- | --- | --- | --- | --- | --- | --- | --- | --- | --- |
| Included (In) or excluded due to death (Ex-D) or other reasons (Ex-O) | In | Ex-D | | Ex-O | In | Ex-D | Ex-O | In | Ex-D | Ex-O | In | Ex-D | | Ex-O | In | Ex-D | Ex-O | | | In | | Ex-D | Ex-O | | In | Ex-D | Ex-O |
|  | **0-11 months** | | | | | | | | | | | | | | | | | | | | | | | | | | |
| N | **324** | **16** | **60** | | **600** | **13** | **113** | **265** | **28** | **81** | **598** | **29** | **46** | | **636** | **2** | | **34** | | | **530** | **5** | **15** | **455** | | **13** | **165** |
| Age in months (mean) | 7.8 | 7.7 | 8.0 | | 7.3 | 7.2 | 7.3 | 6.3 | 7.0 | 6.8 | 6.6 | 5.4† | 6.8 | | 6.8 | 6.0 | | 7.6 | | | 7.2 | 5.0 | 7.7 | 6.1 | | 4.9 | 6.3 |
| % Female | 42.0 | 43.8 | 36.7 | | 45.6 | 46.2 | 42.5 | 38.1 | 42.9 | 43.2 | 40.3 | 37.9 | 47.8 | | 44.2 | 0 | | 52.9 | | | 39.1 | 40.0 | 46.7 | 46.4 | | 38.5 | 46.1 |
| % Caretaker completed primary school | 5.3 | 0 | 13.3† | | 16.6 | 23.1 | 13.3 | 21.8 | 28.6 | 21.0 | 53.9 | 51.7 | 52.2 | | 60.7 | 0 | | 52.9 | | | 76.2 | 60.0 | 86.7 | 17.4 | | 7.7 | 15.8 |
| Mean wealth quintile | 2.0 | 1.8 | 1.7 | | 2.1 | 1.6 | 1.9 | 2.0 | 1.6 | 1.8 | 2.0 | 2.1 | 1.7 | | 1.9 | 1.5 | | 1.9 | | | 2.1 | 1.8 | 2.1 | 1.9 | | 1.3 | 1.7 |
| No. in household (mean) | 28.8 | 33.3 | 23.5 | | 17.3 | 11.9† | 14.7† | 6.8 | 6.6 | 6.7 | 5.6 | 5.6 | 5.7 | | 6.5 | 6.5 | | 5.7 | | | 6.0 | 6.8 | 6.3 | 9.7 | | 8.4 | 8.4 |
| % Access to improved water* | 40.7 | 31.3 | 45.0 | | 66.1 | 30.8† | 61.9 | 29.4 | 35.7 | 23.5 | 9.7 | 13.8 | 13.0 | | 47.5 | 100 | | 70.6† | | | 98.7 | 100 | 100 | 0.9 | | 0 | 1.8 |
| HAZ at baseline | -0.86 | -1.97† | -0.67 | | -0.53 | -0.73 | -0.59 | -1.24 | -2.12† | -0.81 | -0.96 | -2.06† | -0.78 | | -1.01 | -2.66† | | -1.09 | | | -1.05 | -2.34† | -1.30 | -1.64 | | -3.00† | -1.65 |

|  | Basse,  The Gambia | | | | Bamako,  Mali | | | Manhiça, Mozambique | | | Kisumu,  Kenya | | | | Kolkata,  India | | | | Mirzapur,  Bangladesh | | | | | | Karachi (Bin Qasim Town) Pakistan | | |
| --- | --- | --- | --- | --- | --- | --- | --- | --- | --- | --- | --- | --- | --- | --- | --- | --- | --- | --- | --- | --- | --- | --- | --- | --- | --- | --- | --- |
| Included (In) or excluded due to death (Ex-D) or other reasons (Ex-O) | In | Ex-D | | Ex-O | In | Ex-D | Ex-O | In | Ex-D | Ex-O | In | Ex-D | | Ex-O | In | Ex-D | Ex-O | | | In | | Ex-D | Ex-O | | In | Ex-D | Ex-O |
|  | **12-23 months** | | | | | | | | | | | | | | | | | | | | | | | | | | |
| N | 354 | 19 | 82 | | 570 | 6 | 106 | 128 | 19 | 48 | 359 | 14 | 37 | | 558 | 0 | | 30 | | | 463 | 0 | 13 | 309 | | 2 | 88 |
| Age in months (mean) | 16.9 | 17.2 | 17.1 | | 16.9 | 17.2 | 16.0† | 16.0 | 16.6 | 16.8 | 16.4 | 16.4 | 17.3 | | 16.6 | - | | 17.5 | | | 16.7 | - | 14.5† | 16.5 | | 19.0 | 16.2 |
| % Female | 44.6 | 36.8 | 52.4 | | 42.8 | 50.0 | 50.0 | 47.7 | 52.6 | 29.2† | 44.9 | 50.0 | 48.6 | | 45.2 | - | | 23.3† | | | 43.8 | - | 46.2 | 42.4 | | 50.0 | 38.6 |
| % Caretaker completed primary school | 4.8 | 5.3 | 11.0† | | 13.9 | 16.7 | 15.1 | 20.5 | 15.8 | 17.0 | 54.9 | 35.7 | 56.8 | | 54.7 | - | | 60.0 | | | 74.3 | - | 92.3 | 16.8 | | 0 | 6.8† |
| Mean wealth quintile | 2.0 | 1.3† | 1.9 | | 2.1 | 2.0 | 1.9 | 1.9 | 1.4 | 2.2 | 1.9 | 1.9 | 2.0 | | 1.8 | - | | 1.8 | | | 1.8 | - | 2.4 | 1.9 | | 2.0 | 1.6† |
| No. in household (mean) | 27.6 | 25.2 | 28.1 | | 17.3 | 18.5 | 15.7 | 6.9 | 6.4 | 7.3 | 5.2 | 4.3 | 5.0 | | 6.2 | - | | 6.2 | | | 5.8 | - | 7.2 | 9.4 | | 8.5 | 8.6 |
| % Access to improved water* | 45.2 | 52.6 | 42.7 | | 64.9 | 50.0 | 65.1 | 28.1 | 26.3 | 35.4 | 8.4 | 7.1 | 5.4 | | 45.9 | - | | 53.3 | | | 99.2 | - | 100 | 0 | | 0 | 2.3† |
| HAZ at baseline | -1.32 | -2.34† | -1.40 | | -1.06 | -2.45† | -1.29 | -1.61 | -2.87† | -1.87 | -1.53 | -2.72† | -1.62 | | -1.51 | - | | -1.35 | | | -1.33 | - | -1.60 | -2.20 | | -3.62 | -2.52 |

|  | Basse,  The Gambia | | | | Bamako,  Mali | | | Manhiça, Mozambique | | | Kisumu,  Kenya | | | | Kolkata,  India | | | | Mirzapur,  Bangladesh | | | | | | Karachi (Bin Qasim Town) Pakistan | | |
| --- | --- | --- | --- | --- | --- | --- | --- | --- | --- | --- | --- | --- | --- | --- | --- | --- | --- | --- | --- | --- | --- | --- | --- | --- | --- | --- | --- |
| Included (In) or excluded due to death (Ex-D) or other reasons (Ex-O) | In | Ex-D | | Ex-O | In | Ex-D | Ex-O | In | Ex-D | Ex-O | In | Ex-D | | Ex-O | In | Ex-D | Ex-O | | | In | | Ex-D | Ex-O | | In | Ex-D | Ex-O |
|  | **24-59 months** | | | | | | | | | | | | | | | | | | | | | | | | | | |
| N | 133 | 4 | 37 | | 552 | 3 | 69 | 71 | 4 | 37 | 349 | 9 | 35 | | 295 | 0 | | 13 | | | 353 | 2 | 13 | 175 | | 1 | 50 |
| Age in months (mean) | 31.2 | 37.5 | 33.2 | | 35.7 | 33.7 | 34.9 | 34.5 | 33.5 | 32.6 | 36.3 | 34.0 | 35.5 | | 35.4 | - | | 39.5 | | | 34.9 | 30.0 | 34.9 | 35.6 | | 29.0 | 33.1 |
| % Female | 52.6 | 50.0 | 37.8 | | 44.8 | 33.3 | 49.3 | 47.9 | 25.0 | 35.1 | 44.1 | 66.7 | 48.6 | | 39.7 | - | | 46.2 | | | 42.5 | 0 | 38.5 | 38.3 | | 100 | 42.0 |
| % Caretaker completed primary school | 9.0 | 0 | 10.8 | | 15.2 | 0 | 11.6 | 25.7 | 0 | 16.2 | 55.9 | 55.6 | 54.3 | | 60.3 | - | | 61.5 | | | 73.7 | 100 | 69.2 | 18.3 | | 0 | 22.0 |
| Mean wealth quintile | 2.1 | 1.8 | 2.2 | | 2.1 | 1.7 | 1.9 | 2.0 | 0.8 | 2.2 | 2.1 | 1.8 | 2.1 | | 1.8 | - | | 1.2 | | | 2.0 | 1.0 | 1.9 | 1.7 | | 2.0 | 1.7 |
| No. in household (mean) | 26.2 | 27.8 | 22.3 | | 19.1 | 11.7 | 16.7 | 6.3 | 5.8 | 6.0 | 5.3 | 5.8 | 5.4 | | 5.9 | - | | 5.1 | | | 5.6 | 3.0 | 4.7 | 9.3 | | 8.0 | 9.0 |
| % access to improved water* | 38.4 | 75.0 | 21.6 | | 68.1 | 100 | 50.7† | 26.8 | 50.0 | 43.2 | 9.7 | 0 | 14.3 | | 48.8 | - | | 46.2 | | | 99.4 | 100 | 100 | 0.6 | | 0 | 0 |
| HAZ at baseline | -1.75 | -2.84 | -2.35† | | -1.17 | -3.32† | -1.43 | -1.54 | -3.3† | -1.5 | -1.63 | -3.6† | -1.9 | | -1.71 | - | | -1.95 | | | -1.49 | -1.28 | -1.65 | -2.47 | | -6.73† | -3.10† |

Comparing those included versus those excluded due to death, and those included versus those excluded due to other reasons: † statistically significant difference found by t-test for means, chi-square test for proportions, or Fisher exact test for cell sizes < 5 (p<0·05).

* Improved water is defined as: the main source of drinking water for the household is either piped into house or yard, public tap, tube well (deep or shallow), covered well, protected spring, rainwater, or borehole, is accessible within 15 minutes roundtrip, and is available daily.

**Table S2: Change in length/height for age z score (ΔHAZ) between enrollment and follow-up ~60 days after an episode of moderate-to-severe diarrhea, by site and age group**

|  | **0-11 months** | | **12-23 months** | | **24-59 months** | | | |
| --- | --- | --- | --- | --- | --- | --- | --- | --- |
| **Mean (95% confidence interval):** | | | | | | | | |
| **Basse, The Gambia** | Cases=324 | | Cases=354 | | Cases=133 | | | |
| Enrollment HAZ | -0.86 | (-1.00, -0.73) | -1**_._**32 | (-1**_._**44, -1^.^20) | -1**_._**75 | (-1**_._**98, -1**_._**51) | | |
| Follow-up HAZ | -1**_._**14 | (-1**_._**32, -0.96) | -1**_._**60 | (-1**_._**73, -1**_._**47) | -1**_._**82 | | (-2**_._**07, -1**_._**58) | |
| ΔHAZ | -0.28 | (-0.38, -0.18)§ | -0.28 | (-0.36, -0.21)§ | -0.08 | | (-0.14, -0.02) ‡ | |
| **Bamako, Mali** | Cases=600 | | Cases=570 | | Cases=552 | | | |
| Enrollment HAZ | -0.53 | (-0.68, -0.38) | -1**_._**06 | (-1**_._**17, -0.95) | -1**_._**17 | | (-1**_._**28, -1**_._**05) | |
| Follow-up HAZ | -0.84 | (-1.00, -0.68) | -1**_._**14 | (-1**_._**26, -1**_._**01) | -1^.^09 | | (-1**_._**20, -0.98) | |
| ΔHAZ | -0.31 | (-0.36, -0.25)§ | -0.08 | (-0.13, -0.03)§ | 0.08 | | (0.05, 0.11)§ | |
| **Manhiça, Mozambique** | Cases=265 | | Cases=128 | | Cases=71 | | | |
| Enrollment HAZ | -1**_._**24 | -1**_._**46, -1**_._**01 | -1**_._**61 | -1**_._**79, -1**_._**42 | -1**_._**54 | | | -1**_._**81, -1**_._**26 |
| Follow-up HAZ | -1**_._**45 | -1**_._**60, -1**_._**31 | -2**_._**05 | -2**_._**24, -1**_._**85 | -1**_._**68 | | | -1**_._**94, -1**_._**41 |
| ΔHAZ | -0.22 | -0.33, -0.10§ | -0.44 | -0.50, -0.39§ | -0.14 | | | -0.24, -0.04‡ |
| **Kisumu, Kenya** | Cases=598 | | Cases=359 | | Cases=349 | | | |
| Enrollment HAZ | -0.96 | (-1**_._**08, -0.83) | -1**_._**53 | (-1**_._**72, -1**_._**35) | -1**_._**63 | | (-1**_._**86, -1**_._**41) | |
| Follow-up HAZ | -1^.^32 | (-1**_._**46, -1^.^18) | -1**_._**83 | (-2**_._**01, -1**_._**66) | -1**_._**77 | | (-1**_._**99, -1**_._**54) | |
| ΔHAZ | -0.36 | (-0.43, -0.29)§ | -0.30 | (-0.36, -0.24)§ | -0.13 | | (-0.16, -0.11)§ | |
| **Kolkata, India** | Cases=636 | | Cases=558 | | Cases=295 | | | |
| Enrollment HAZ | -1**_._**01 | -1**_._**09, -0.93 | -1**_._**51 | -1**_._**70, -1**_._**31 | -1**_._**71 | | -1**_._**92, -1.49 | |
| FU HAZ | -1**_._**30 | -1**_._**39, -1**_._**20 | -1**_._**64 | -1**_._**78, -1**_._**49 | -1**_._**75 | | -1**_._**93, -1**_._**56 | |
| ΔHAZ | -0.29 | -0.36, -0.22§ | -0.13 | -0.19, -0.07§ | -0.04 | | -0.08, 0.006‡ | |
| **Mirzapur, Bangladesh** | Cases=530 | | Cases=463 | | Cases=353 | | | |
| Enrollment HAZ | -1**_._**05 | -1**_._**18, -0.93 | -1**_._**33 | -1**_._**45, -1**_._**22 | -1**_._**49 | | -1**_._**57, -1_._41 | |
| FU HAZ | -1**_._**33 | -1**_._**51, -1**_._**14 | -1**_._**51 | -1_._65, -1_._37 | -1_._64 | | -1_._73, -1_._56 | |
| ΔHAZ | -0.28 | -0.41, -0.14§ | -0.18 | -0.22, -0.13§ | -0.15 | | -0.19, -0.11§ | |
| **Karachi (Bin Qasim Town), Pakistan** | Cases=455 | | Cases=309 | | Cases=175 | | | |
| Enrollment HAZ | -1_._64 | -1_._79, -1_._49 | -2_._20 | -2_._42, -1_._99 | -2_._47 | | -2_._68, -2_._25 | |
| FU HAZ | -1_._90 | -2_._07, -1_._73 | -2_._39 | -2_._62, -2_._17 | -2_._52 | | -2_._73, -2_._32 | |
| ΔHAZ | -0.26 | -0.33, -0.19§ | -0.19 | -0.24, -0.14§ | -0.06 | | -0.10, -0.02‡ | |
| **Combined 7 sites** | Cases=3,408 | | Cases=2,741 | | Cases=1,928 | | | |
| Enrollment HAZ | -1_._01 | -1_._09, -0.93 | -1_._45 | -1_._53, -1_._36 | -1_._57 | | -1_._68, -1_._46 | |
| FU HAZ | -1_._30 | -1_._38, -1_._22 | -1_._64 | -1_._74, -1_._54 | -1_._62 | | -1_._75, -1_._49 | |
| ΔHAZ | -0.29 | -0.32, -0.26§ | -0.19 | -0.22, -0.16§ | -0.05 | | -0.09, -0.02§ | |

HAZ denotes height-for-age z score; ΔHAZ denotes HAZ at the follow-up visit minus HAZ at enrollment.

Change in HAZ (ΔHAZ) between enrollment and follow-up was compared by t-test: £p=0.01 to <0.05; ‡p=0.0001 to 0.009; §p<0.0001

**Table S3: Number (%) of episodes of watery diarrhea, dysentery, and *Shigella*-positive dysentery for which antibiotics were prescribed, by type of antibiotic and site, among children 0-59 months of age.**

|  | **Gambia** | **Mali** | **Mozambique** | **Kenya** | **India** | **Bangladesh** | **Pakistan** | **Total** |
| --- | --- | --- | --- | --- | --- | --- | --- | --- |
| **Watery diarrhea** | **n=635** | **n=1,507** | **n=380** | **n=1,154** | **n=1,309** | **n=321** | **n=745** | **n=6,051** |
| **Any antibiotic** | 501 (78.9) | 1,389 (92.2) | 310 (81.6) | 848 (73.5) | 1,139 (87.0) | 248 (77.3) | 45 (6.0) | 4,480 (74.0) |
| **Ciprofloxacin** | 8 (1.3) | 0 | 3 (0.8) | 2 (0.2) | 923 (70.5) | 14 (4.4) | 29 (3.9) | 979 (16.2) |
| **Cephalosporins-3^rd^ generation** | 0 | 180 (11.9) | 7 (1.8) | 9 (0.8) | 29 (2.2) | 15 (4.7) | 2 (0.3) | 242 (4.0) |
| **Azithromycin** | 0 | 2 (0.1) | 1 (0.3) | 0 | 5 (0.4) | 161 (50.2) | 0 | 169 (2.8) |
| **Pivmecillinam** | 0 | 0 | 0 | 1 (0.1) | 1 (0.1) | 0 | 0 | 2 (0.03) |
| **Cephalosporins-1^st^ or 2^nd^ generation** | 0 | 171 (11.4) | 0 | 0 | 7 (0.5) | 0 | 1 (0.1) | 179 (3.0) |
| **Trimethoprim/sulfamethoxazole** | 336 (52.9) | 1,019 (67.6) | 72 (19.0) | 682 (59.1) | 137 (10.5) | 23 (7.2) | 0 | 2,269 (37.5) |
| **Chloramphenicol** | 56 (8.8) | 5 (0.3) | 123 (32.4) | 3 (0.3) | 1 (0.1) | 0 | 1 (0.1) | 189 (3.1) |
| **Gentamycin** | 16 (2.5) | 38 (2.5) | 133 (35.0) | 79 (6.9) | 0 | 3 (0.9) | 0 | 269 (4.5) |
| **Metronidazole** | 42 (6.6) | 591 (39.2) | 15 (4.0) | 106 (9.2) | 617 (47.1) | 0 | 3 (0.4) | 1,374 (22.7) |
| **Nalidixic acid** | 0 | 0 | 4 (1.1) | 20 (1.7) | 6 (0.5) | 0 | 2 (0.3) | 32 (0.5) |
| **Penicillin** | 38 (6.0) | 5 (0.3) | 54 (14.2) | 60 (5.2) | 1 (0.1) | 0 | 0 | 158 (2.6) |
| **Amoxicillin** | 52 (8.2) | 62 (4.1) | 70 (18.4) | 50 (4.3) | 11 (0.8) | 0 | 7 (0.9) | 252 (4.2) |
| **Ampicillin** | 21 (3.3) | 3 (0.2) | 103 (27.1) | 3 (0.3) | 0 | 2 (0.6) | 2 (0.3) | 134 (2.2) |
| **Erythromycin** | 9 (1.4) | 33 (2.2) | 4 (1.1) | 42 (3.6) | 4 (0.3) | 44 (13.7) | 0 | 136 (2.3) |
| **Dysentery** | **n=176** | **n=215** | **n=84** | **n=152** | **n=180** | **n=1,025** | **n=194** | **n=2,026** |
| **Any antibiotic** | 151 (85.8) | 198 (92.1) | 71 (84.5) | 127 (83.6) | 171 (95.0) | 1,021 (99.6) | 155 (79.9) | 1,894 (93.5) |
| **WHO-recommended antibiotic*** | 11 (6.3) | 31 (14.4) | 1 (1.2) | 25 (16.5) | 144 (80.0) | 910 (88.8) | 155 (79.9) | 1,277 (63.0) |
| **Ciprofloxacin** | 10 (5.7) | 1 (0.5) | 1 (1.2) | 21 (13.8) | 140 (77.8) | 887 (86.5) | 153 (78.9) | 1,213 (59.9) |
| **Cephalosporins-3^rd^ generation** | 0 | 31 (14.4) | 0 | 4 (2.6) | 4 (2.2) | 7 (0.7) | 2 (1.0) | 48 (2.4) |
| **Azithromycin** | 0 | 0 | 0 | 0 | 0 | 19 (1.9) | 0 | 19 (0.9) |
| **Pivmecillinam** | 0 | 0 | 0 | 0 | 0 | 3 (0.3) | 0 | 3 (0.2) |
| **Other antibiotic** | 141 (80.1) | 168 (78.1) | 70 (83.3) | 102 (67.1) | 27 (15.0) | 134 (13.1) | 1 (0.5) | 620 (30.6) |
| **Cephalosporins-1^st^ or 2^nd^ generation** | 0 | 27 (12.6) | 1 (1.2) | 0 | 0 | 0 | 0 | 28 (1.4) |
| **Trimethoprim/sulfamethoxazole** | 98 (55.7) | 140 (65.1) | 10 (11.9) | 75 (49.3) | 24 (13.3) | 110 (10.7) | 0 | 457 (22.6) |
| **Chloramphenicol** | 20 (11.4) | 0 | 16 (19.1) | 0 | 1 (0.6) | 0 | 0 | 37 (1.8) |
| **Gentamycin** | 3 (1.7) | 7 (3.3) | 3 (3.6) | 7 (4.6) | 0 | 0 | 0 | 20 (1.0) |
| **Metronidazole** | 14 (8.0) | 94 (43.7) | 0 | 27 (17.8) | 100 (55.6) | 0 | 0 | 235 (11.6) |
| **Nalidixic acid** | 0 | 0 | 46 (54.8) | 18 (11.8) | 1 (0.6) | 0 | 1 (0.5) | 66 (3.3) |
| **Penicillin** | 5 (2.8) | 0 | 1 (1.2) | 5 (3.3) | 0 | 0 | 0 | 11 (0.5) |
| **Amoxicillin** | 11 (6.3) | 8 (3.7) | 7 (8.3) | 1 (0.7) | 1 (0.6) | 0 | 0 | 28 (1.4) |
| **Ampicillin** | 5 (2.8) | 0 | 4 (4.8) | 0 | 0 | 0 | 0 | 9 (0.4) |
| **Erythromycin** | 2 (1.1) | 3 (1.4) | 0 | 3 (2.0) | 0 | 2 (0.2) | 0 | 10 (0.5) |
| ***Shigella* positive dysentery** | **n=50** | **n=15** | **n=8** | **n=37** | **n=47** | **n=515** | **n=59** | **n=731** |
| **Any antibiotic** | 45 (90.0) | 14 (93.3) | 8 (100) | 30 (81.1) | 45 (95.7) | 514 (99.8) | 55 (93.2) | 711 (97.3) |
| **WHO-recommended antibiotic** | 7 (14.0) | 4 (26.7) | 0 | 9 (24.3) | 40 (85.1) | 468 (90.9) | 55 (93.2) | 583 (79.8) |
| **Ciprofloxacin** | 7 (14.0) | 0 | 0 | 7 (18.9) | 39 (83.0) | 453 (88.0) | 55 (93.2) | 561 (76.7) |
| **Cephalosporins-3^rd^ generation** | 0 | 4 (26.7) | 0 | 2 (5.4) | 1 (2.1) | 4 (0.8) | 0 | 11 (1.5) |
| **Azithromycin** | 0 | 0 | 0 | 0 | 0 | 14 (2.7) | 0 | 14 (1.9) |
| **Pivmecillinam** | 0 | 0 | 0 | 0 | 0 | 1 (0.2) | 0 | 1 (0.1) |
| **Other antibiotic** | 37 (75.5) | 10 (66.6) | 8 (100) | 20 (55.6) | 5 (10.6) | 46 (8.9) | 0 | 126 (17.3) |
| **Cephalosporins-1^st^ or 2^nd^ generation** | 0 | 2 (13.3) | 0 | 0 | 0 | 0 | 0 | 2 (0.3) |
| **Trimethoprim/sulfamethoxazole** | 30 (60.0) | 9 (60.0) | 0 | 15 (40.5) | 5 (10.6) | 46 (8.9) | 0 | 105 (14.4) |
| **Chloramphenicol** | 4 (8.0) | 0 | 0 | 0 | 1 (2.1) | 0 | 0 | 5 (0.7) |
| **Gentamycin** | 0 | 1 (6.7) | 0 | 1 (2.7) | 0 | 0 | 0 | 2 (0.3) |
| **Metronidazole** | 4 (8.0) | 5 (33.3) | 0 | 8 (21.6) | 29 (61.7) | 0 | 0 | 46 (6.3) |
| **Nalidixic acid** | 0 | 0 | 7 (87.5) | 5 (13.5) | 1 (2.1) | 0 | 0 | 13 (1.8) |
| **Penicillin** | 2 (4.0) | 0 | 1 (12.5) | 0 | 0 | 0 | 0 | 3 (0.4) |
| **Amoxicillin** | 2 (4.0) | 0 | 1 (12.5) | 1 (2.7) | 0 | 0 | 0 | 4 (0.6) |
| **Ampicillin** | 0 | 0 | 0 | 0 | 0 | 0 | 0 | 0 |
| **Erythromycin** | 1 (2.0) | 0 | 0 | 0 | 0 | 1 (0.2) | 0 | 2 (0.3) |

*WHO-recommended antibiotics for dysentery, which include ciprofloxacin, third generation cephalosporins, azithromycin, and pivmecillinam
